# Epidemiology, trend and in-hospital outcome of traumatic spinal injuries due to road traffic accidents

**DOI:** 10.12669/pjms.38.3.5288

**Published:** 2022

**Authors:** Mubarak Ali Algahtany

**Affiliations:** 1 Dr. Mubarak Ali Algahtany, MD, FRCSC. Division of Neurosurgery, Department of Surgery, College of Medicine, King Khalid University, Abha 62512-2291, KSA

**Keywords:** Traumatic spinal injury, Spinal cord injury, Road traffic accident, epidemiology

## Abstract

**Objectives:**

This study aimed to provide information on the epidemiology, trend, associated traumatic spinal cord injury (TSCI) and non-spinal injuries, and risk factors affecting the in-hospital outcome of RTA-related traumatic spinal injury (TSI) over the past decade in Aseer province, Saudi Arabia.

**Methods:**

In this retrospective study, we included all RTA-related traumatic injuries (5797) admitted to ACH from 1st January 2010 to 31 December 2019, from which 810 cases were TSIs. The cases were identified through the hospital database registry.. Descriptive analysis was performed for gender, age, level of spinal injury, admission day, type of care unit, associated injuries, presence of TSCI and discharge category.

**Results:**

TSIs accounted for 13.97% of RTA-related injuries with a predominantly male population. The patients had a mean age of 30.7 years, 46.67 % of victims were between 19 to 30-years old. There was a significant decrease in the number of RTA-related TSIs over the study period. Lumbar and cervical injuries were more prevalent (32.47 and 31.36 %, respectively). Most (73.58 %) TSIs occurred during working days, and 6.54% required critical care admission. Associated non-spinal injuries occurred in 50.25% and TSCI in 6.91% of patients. The in-hospital mortality was 5.18%. The Age, level of spine injury, need for critical care, associated injuries, and TSCI significantly affected the likelihood of improvement.

**Conclusion:**

The latest government initiatives to reduce RTA like speed limits and waring of seat belts have resulted in a concordant decline in TSI incidence with good consistency between police and health registration data

## INTRODUCTION

 Epidemiological data on traumatic spinal injury (TSI) in the Kingdom of Saudi Arabia (KSA) is limited. Most of the previous studies on TSI from KSA have focused on the traumatic spinal cord injury (TSCI) -which represents only a tiny fraction of the TSI-, included all etiologies, and were short-term. Road traffic accident (RTA) is a significant cause of both TSI and TSCI globally while being the principle mechanism of both injuries in KSA.^1^-^3^ Dedicated studies of RTA-related TSI are very scarce internationally and virtually non-existing in KSA. Traffic injury, however, is a real problem in the country, which has one of the highest globally reported figures of RTA due to four-wheeled vehicles and one of the world’s highest prevalence of TSCI resulting from RTA.^4^ Accordingly, the country strictly implemented tougher traffic regulations over the past decade to reduce the societal burdens of RTA, including health-related ones. Yet, the effectiveness of these measures on the epidemiology of TSI has not been studied.

 Furthermore, most of the previous studies on TSI have been mainly from the country’s capital city, nonetheless a regional difference of TSI epidemiology is well documented.^1^ This variability is likely to be more evident when studying RTA-related TSI due to the expected influential effect of regional differences in demographic, culture, and terrain on the RTAs themselves. Therefore, it is imperative to have long-term, provincial studies of the whole group of TSI, based on which regional-based effective policies are made.

## METHODS

 This retrospective medical record study which was conducted at Aseer region, KSA. The province is 76,693 square kilometers (29,611 sq mi), southwest of KSA, occupies 3.7% of its land, and is the country’s fourth most populated province with 2308329 residents in 2019.^5^ The study was conducted in the only tertiary hospital in the province-Aseer Central Hospital (ACH) in the provincial capital city of Abha.

 In this study, we included all RTA-related traumatic injuries (5797) admitted to ACH from 1st January 2010 to 31st December 2019, from which 810 cases were TSIs. The cases were identified through the hospital database registry. Descriptive analysis was performed for gender, age, level of spinal injury, admission day, type of care unit, associated injuries, presence of TSCI, and discharge category. The patients were categorized based on their discharge category into either improved (discharged home following improvement) or not improved (expired or transferred to other facilities for further care).

### Statistical Analysis

After coding, statistical analysis was conducted using IBM SPSS software SPSS statistical system package (version 23, Armonk, NY, USA) to analyze the data. A simple descriptive analysis was used to describe the association between variables. The Chi-Square test was applied to determine the significant relationships (*p* < 0.05). A univariate logistic regression model was fitted through Maximum Likelihood (ML) procedure to evaluate the strength of association between the independent variables (gender, age, level of spinal injury, admission day, type of care unit, associated injuries, and presence of TSCI) and the patients’ improvement. Results are expressed as percentages and crude odds ratios and their 95% confidence interval (95% CI).

### Institutional Review Board Approval

The study was done according to the guidelines of the Declaration of Helsinki and approved by the Research Ethics Committee at King Khalid University (ECM# 2021-4001, 10-03-2021)

## RESULTS

**Fig.1:**
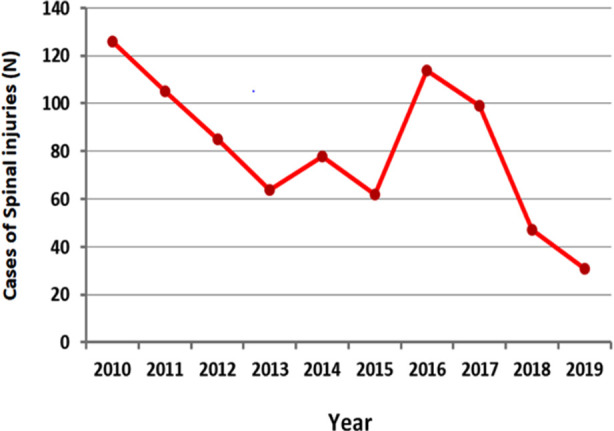
Annual distribution for the number of traumatic spinal injuries due to road traffic accidents.

 Through the study duration, a total of 5797 patients presented with traumas attributed to road traffic accidents (RTA-related traumatic injuries), from which 810 (13.97%) patients were traumatic spinal injuries (RTA-related TSIs) (Table-I). The percentage ranged from 9.20% (in 2015) to 15.91% (in 2011). The TSI number started to decrease from the beginning, with two spikes in 2014 and 2017, and subsequently continued to decline until the end of the study (Fig.1).

**Table-I:** Distribution of traumatic spinal injuries in relation to the total number of all traumatic injuries due to road traffic accidents.

Year	Total number of traumatic cases	Traumatic spinal injury cases		Chi-Square value

		n	%†	
2010	818	126	15.40	18.76*
2011	660	105	15.91	
2012	603	85	14.09	
2013	504	64	12.70	
2014	570	78	13.68	
2015	674	62	9.20	
2016	787	114	14.49	
2017	631	99	15.69	
2018	336	47	13.99	
2019	214	30	14.02	
TOTAL	5797	810	13.97	

* Chi-Square value significantly differs at *p* = 0. 027.

†The percentage of traumatic spinal injuries due to road traffic accidents was calculated based on the total number of all traumatic injuries due to the exact mechanism.

**Fig.2:**
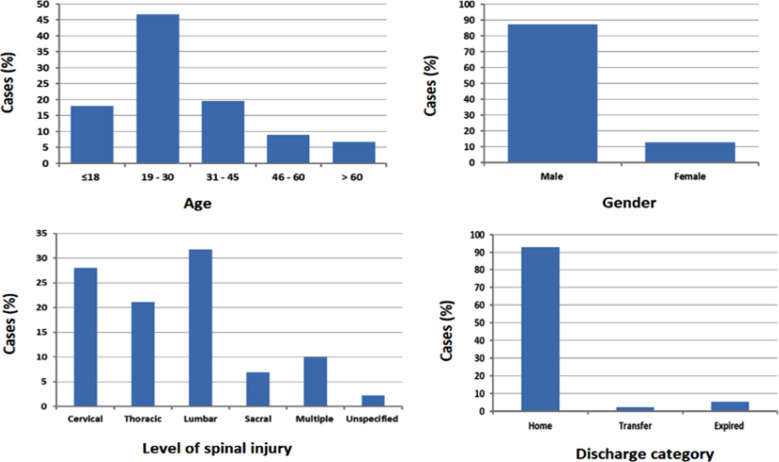
Distribution of traumatic spinal injuries due to road traffic accidents in relation to age, gender, level of spinal injury, and discharge category.

 The majority (87.28 %) of patients were male versus 12.72 % as female patients with a male to female ratio of 6.86: 1 (Table-II and Fig.2). The mean age of the cohort was 30.7 ± 0.56. Young adults (19 – 30-year-old) constituted the majority (46.67 %) of patients, while those aged over 60-year-old were the least (6.79 %). The majority (73.58 %) of admissions occurred during working days.

**Table-II:** Epidemiological characteristics of traumatic spinal injuries due to road traffic accidents.

Factors	Class	Cases (n)	Cases (%)
Gender			
	Male	707	87.28
	Female	103	12.72
	Ratio	6.86: 1	
* Age (years)	30.7 ± 0.56		
	≤18	146	18.02
	19 – 30	378	46.67
	31 – 45	159	19.63
	46 – 60	72	8.89
	> 60	55	6.79
Level of spinal injury			
	Cervical	227	28.03
	Thoracic	171	21.11
	Lumbar	257	31.73
	Sacral	56	6.91
	Multiple	81	10.0
	Unspecified	18	2.22
Admission day			
	Weekend	214	26.42
	Weekday	596	73.58
Critical care unit admission			
	No	757	93.46
	Yes	53	6.54
Associated non-spinal injuries			
	No	403	49.75
	Yes	407	50.25
Traumatic spinal cord injury			
	No	754	93.09
	Yes	56	6.91
Discharge category			
	Home	751	92.72
	Transfer	17	2.10
	Expired	42	5.18

* Age is illustrated as mean ± standard error.

 Regarding the level of spinal injury, lumbar and cervical injuries were more prevalent (31.73% and 28.03 %, respectively), followed by thoracic, multiple levels, and then sacral injury, where the respective values were 21.11%, 10.0%, and 6.91% (Table-II and Fig.2). Overall, 6.54% of patients required critical care unit admission for more intensive care. Half (50.25%) of RTA-related TSIs had associated non-spinal injury and 6.91% had TSCI. Concerning the discharge category, 92.72% of patients improved and discharged home, 2.10% were transferred to other specialized facilities for further management, and 5.18% expired during their hospital stay (Table-II and Fig.2).

 The odd ratios and outputs of logistic regression model for risk factors affecting TSI’s improvement likelihood are illustrated in Table-III. The possibility of RTA-related TSI improvement in females was higher than in males but was not statistically significant (Odd ratio = 1.24, *p* = 0.561). Furthermore, patients of age 31–45, 46–60, and over 60-year-old have a lower likelihood of improvement compared with younger patients (Odd ratios = 0.25, 0.15 and 0.14, *p* = 0.015, 0.001, and 0.006, respectively). The injury during weekday vs. weekend did not affect the probability of improvement of TSI (Odd ratio = 1.2, *p* = 0.46).

**Table-III:** Logistic regression for factors affecting the likelihood of improvement of traumatic spinal injuries due to road traffic accidents.

Factors	Class	Odd ratio	95% CI	p -value
Gender				
	Male	Reference		
	Female	1.24	0.60 – 2.55	0.561
Age (years)				
	≤18	Reference		
	19 – 30	0.46	0.17 – 1.16	0.107
	31 – 45	0.25	0.08 – 0.77	0.015*
	46 – 60	0.15	0.05 – 0.39	0.001*
	> 60	0.14	0.06 – 0.43	0.006*
Level of spinal injury				
	Unspecified	Reference		
	Cervical	0.98	0.27 – 3.61	0.897
	Thoracic	4.07	0.97 – 16.24	0.054
	Lumber	8.37	1.90 – 36.77	0.005*
	Sacral	5.40	1.21 – 35.33	0.039*
	Multiple	5.21	0.96 – 28.26	0.057
Admission day				
	Working days	Reference		
	Weekend	0.83	0.51 - 1.36	0.463
Critical care unit admission				
	No	Reference		
	Yes	0.04	0.03 – 0.08	0.001*
Presence of associated injuries				
	No	Reference		
	Yes	0.33	0.21 – 0.57	0.001*
Traumatic spinal cord injury				
	No	Reference		
	Yes	0.32	0.16 – 0.69	0.003*

* Significant level was considered at p < 0.05.

 Lumbar and sacral spinal injury had a lower likelihood for improvement when compared with unspecified spinal injury (Odd ratios = 8.37 and 5.40, *p* = 0.003 and 0.039, respectively). The group which needed ICU admission had a lesser likelihood to improve (Odd ratio = 0.04, *p* = 0.001). The presence of associated injury lowered the likelihood of improvement compared to patients with only spinal injury (Odd ratio = 0.33, *p* = 0.001) and similarly the patients with TSCI had a lower probability for improvement (Odd ratio = 0.32, *p* = 0.003).

## DISCUSSION

 Though rare, traumatic spinal injuries have significant potential to cause death and devastating disability with an enormous burden on the individual, caregiver, and the healthcare system.^1^,^5^ Polytraumatized patients with TSI have higher mortality and more unsatisfactory outcome compared to patients with no spinal injury.^6^-^14^ The rate of TSI in Saudi Arabia is one of the highest globally (62 TSI per million), with RTA being the primary mechanism of injury.^4^,^12^,^15^

 Aseer region, with only 3.7% of Saudi Arabia’s land and over 2308329 inhabitants, is the most densely populated province in the country. Among the 28 ministry of health hospitals in the province, Aseer Central Hospital is the only tertiary hospital and the only one with specialized spine service. This has not changed over the study period. All traffic accident victims are eligible for treatment in ACH, where all spinal injuries are routinely transferred to it for care. This combination of a densely populated small geographic region with only one tertiary hospital provides a unique opportunity to study the impact of implementing strict traffic regulations on the pattern and trend of RTA-related TSI. Further, it offers the chance to explore the consistency between police and health system records regarding RTA-related injuries.

 This study reveals a significant trend toward decreasing RTA-related TSI over the past decade. This is the first medical study from KSA that proves the efficacy of the recent efforts to reduce health-related consequences of RTA. Further, this is the first study to show the consistency of police and health registration records on RTA-related injuries in KSA. The steady decrease of TSI following the spike in 2017 could be related to the prohibition of mobile use while driving, which was implemented from the beginning of 2018 and enforced by the most advanced camera monitoring system.

 RTA, however, is still a significant cause of morbidity and mortality in Aseer province. In 2019 a total of 25342 accidents occurred in Aseer, causing 603 deaths and 1854 injuries.^5^ This turns into 26 deaths and 80 injuries per 100,000 population. The ratios of accident to death and accident to injury are 40:1, 14:1 respectively, which are higher than international figures.^7^ This study shows that one of each seven RTA-related injuries had TSI, predominantly young males, with 5%, 7% risk of death and TSCI, respectively. The finding in this study of largely young male victims is consistent with all previous reports from Saudi Arabia.^10^-^12^ Having TSCI at a young age carries devastating consequences on the individual. Furthermore, given that the most affected age groups provide the society’s financial security, the societal burden of RTA-related TSI is enormous due to the number of productive years lost plus the direct and indirect care cost. Advanced age appeared in this study as an adverse prognostic factor for improvement, consistent with previous reports.^10^

 This study shows that the majority (73.58%) of RTA-related TSI occurred during the working days likely attributed to the traffic congestion, considering that motor vehicle is the principal transport method in KSA. However, the occurrence of the injury during weekday or weekend did not impact the discharge outcome.

 In our study, 6.54% of patients required a critical care unit admission for more intensive care. This group of patients had more unsatisfactory discharge outcomes than those who did not need intensive care.

 In this study, the lumbar and cervical regions were the most involved levels which concur with national and international previous studies.^3^,^6^,^13^ Our findings of lumbar more than cervical RTA-related spinal injury conform with the same observation by others.^14^,^15^ Some studies, however, have observed that the RTA-related TSI tends to happen more often in the cervical spine.^13^,^16^ This study reveals that half of TSI patients had associated non-spinal injuries and documents their negative impact on the outcome. This is supported by other studies that showed the increased mortality in TSI patients with other non-spinal injuries.^12^,^15^

 The percentage of TSCI in this study was 6.91%, like the 6.6% in a previous report of RTA-related TSI.^20^ It is also close to the 6.7%, 11%, and 11.9 % reported from national and international studies on TSI due to all mechanisms.^15^,^18,22^ Our results show that the presence of TSCI negatively affected the likelihood of RTA-related TSI improvement.

 The 5.18% mortality reported here is better than previously reported from Saudi Arabia (7.6-10.9%).^15^,^16^ This could be due to a decrease in the injury severity related to stricter traffic rules implementation. Previous studies from KSA unanimously showed low percentages of seat belt wearing among RTA victims.^12^,^15^,^16^ The observation that over 93% of patients in this study did not require high dependency care unit supports a decrease in the injury severity. Another explanation is that the injury mechanism in this study is solely RTA instead of all etiologies in previous studies.

## CONCLUSIONS

 Road traffic accidents are still a significant cause of both TSI and TSCI in Saudi Arabia, with young adult males being the primary victims. The latest government initiatives to reduce RTA like speed limit and wearing of seat belts have resulted in a concordant decline in TSI incidence with good consistency between police and health registration data. However, the mortality and TSCI reported in this study call for more efforts to reduce RTA and its related injuries substantially.
